# Specific ion effects enhance local structure in zwitterionic osmolyte solutions†

**DOI:** 10.1039/d5sc00286a

**Published:** 2025-03-17

**Authors:** Kieran J. Agg, Timothy S. Groves, Shurui Miao, Y. K. Catherine Fung, Oliver L. G. Alderman, Thomas F. Headen, Terri-Louise Hughes, Gregory N. Smith, Tristan G. A. Youngs, James P. Tellam, Yao Chen, Susan Perkin, James E. Hallett

**Affiliations:** a Physical and Theoretical Chemistry Laboratory, Department of Chemistry, University of Oxford Oxford OX1 3QZ UK; b ISIS Neutron and Muon Source, Rutherford Appleton Laboratory Didcot OX11 0QX UK; c Department of Chemistry, School of Chemistry, Food and Pharmacy, University of Reading Reading RG6 6AD UK j.e.hallett@reading.ac.uk

## Abstract

Zwitterionic osmolytes are widely known to have a protein-protective effect against high salt concentration, but a mechanistic picture of osmolyte function remains elusive. Here total scattering is used to determine the room temperature liquid structure of two model cytosol solutions containing trimethylglycine (TMG) with either sodium or potassium chloride. H/D isotopic substitution is used to obtain differential neutron scattering cross sections at multiple contrasts in addition to an X-ray structure factor, and an Empirical Potential Structure Refinement (EPSR) simulation is fitted to the experimental data. We reveal the nature of the interaction between TMG molecules and ions in solution, observing binding between cations and the TMG carboxylate group. We observe three key specific ion effects: first, that sodium ions are more tightly localised at the carboxylate group; second, that sodium localisation in turn promotes head-to-head bridging between carboxylate groups when compared to potassium or no added ions, resulting in strong oxygen–oxygen correlations; and third, that sodium ions promote TMG clusters with greater orientational order, more fully shielding the ion but also in turn limiting access to the carboxylate groups for other molecules. These observations have implications for the bioavailability and protein-stabilising effect of osmolytes under changing extracellular salt conditions.

## Abstract 2

Using total scattering and simulations, we describe how different salt species influence zwitterion cluster morphologies in water.

## Graphical abstract

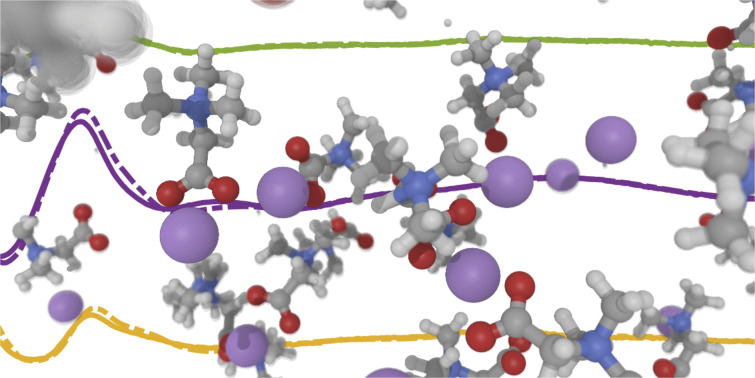

## 1 Introduction

The cellular environment utilises a diverse assortment of ions and molecules to fine-tune biochemical processes. The chemical species accumulated in the cellular fluid, or cytosol, are essential in sustaining osmotic pressure in balance with the surrounding medium, driving the chemical potential of water towards equilibrium on either side of the cell membrane.^1^ In addition to optimising the colligative properties of the cytosol, the chemical makeup of the cellular fluid can also significantly perturb interactions between biomolecules – such as electrostatic and dispersion forces – which are fine-tuned for optimised biological processes.^2^

There is a striking similarity in the molecules that are accumulated across a plethora of organisms. Specifically, zwitterionic molecules including trimethylglycine (TMG), proline (Pro), and trimethylamine *N*-oxide (TMAO) are widely found throughout the biosphere^3–5^ and can all be classified as osmoprotectants, or osmolytes – that is, molecules that provide increased resistance to osmotic stress. While osmotic concentrations of any single osmolyte rarely exceed 100 mOsm/kg in typical physiological environments (where total osmolality ∼ 300 mOsm kg^−1^), *total* osmolality can nevertheless reach Osm kg^−1^ quantities in certain organs.^6^ However, in the most strongly halophilic organisms – those that have evolved to overcome a strong osmotic barrier to life – concentrations of *individual* osmolytes can reach several molar.^7,8^ Additionally, whilst the accumulation of neutral osmolytes such as TMG and of salt are sometimes seen as independent strategies for osmoprotection – which can even be exhibited independently by the same organism, depending on the growth conditions^9^ – some organisms in the most extreme conditions can demonstrate elevated concentrations of both zwitterionic and ionic solutes.^10^

In recent years, various physicochemical properties of these zwitterions have been studied with the aim of understanding how they stabilise biological macromolecules^11^ in addition to contributing to the internal osmotic pressure of the cell. Such studies have included investigating the perturbation of water structure in the presence of these molecules in addition to their local solvation environments,^12^ resulting from their antagonistic dipolar and hydrophobic properties.^13^ Others have proposed that network formation or clustering of the osmolytes themselves plays a key role in determining the thermodynamic stability of proteins in solution.^14^ Despite the depth and breadth of research on their importance in cell function, mechanistic insight of osmolyte function is still lacking.

In addition to providing resistance to osmotic stress or stabilising protein structure, many osmolytes play a key role in metabolic or synthetic processes. For example, TMG contributes to the synthesis of many biological molecules through various metabolic pathways,^15^ either as a co-factor in methylation, or by ultimately degrading to sarcosine or glycine, which are then incorporated into proteins. Any action that compromises the bioavailability of osmolytes can then have a negative impact on cell health or function.^16^ There is therefore an interplay between these competing factors: on the one hand, bioavailability of these molecules is essential for maintaining metabolic processes; on the other, accumulation either throughout the cell or at specific proteins can improve stability. Understanding the solvation environment of biomolecules can thus provide key insight into their function in changing cellular conditions.

Structural determination using neutron and X-ray scattering techniques has been fundamental in the study of water and aqueous solutions,^17^ and has been essential in monitoring perturbations to the structure of water upon the introduction of simple, monovalent ions into a solution.^18–20^ In recent years, this approach has been increasingly used to understand the solvation environment of biomolecules, and there are now numerous studies looking at the effects of osmolytes on the structure of water, such as proline,^21^ TMAO,^22^ urea^23^ and trehalose.^24^ However, there are relatively few studies considering the combined effects of molecular osmolytes and ionic species in a solution,^25^ yet as we have discussed, biological environments contain complex mixtures of solutes, ranging in chemical complexity from inorganic ions to small organic molecules.^9^

Recently, an additional role for zwitterionic osmolytes has also been revealed: that of mediating long range electrostatic interactions.^26^ Adding zwitterions has the reverse effect to adding salt – increasing the range of electrostatic repulsions. As such, zwitterions can prevent protein aggregation by restoring charge repulsions.^27^ Furthermore, recent works have proposed a role for these zwitterionic osmolytes in preventing the salting out of proteins *via* the formation of ion–molecular associates.^28^ In order to unravel the combined role of zwitterionic osmolytes and ionic species in biological cells, a model cytosol solution is required.

Here, we present total scattering measurements performed on aqueous mixtures of the osmolyte, TMG, and a monovalent chloride salt – either potassium or sodium chloride. These measurements reveal the impact of specific ions on various structural motifs within these solutions, such as TMG–ion pairs and TMG–TMG clusters, allowing us to further our understanding of the role of these zwitterionic osmolytes in maintaining biomolecule structure and function.

## 2 Materials and methods

### 2.1 Neutron and X-ray diffraction

A total scattering measurement, whether using neutrons or X-rays, involves the measurement of the differential cross section d*σ*/d*Ω*, a measure of the flux of the scattered radiation at each solid angle around the scattering object. From this quantity it is possible to determine the total structure factor *F*(*Q*) as function of the scattering angle *Q*, the weighted sum of the partial structure factors *S*_*ij*_ present in the sample, as described by eqn (1):1
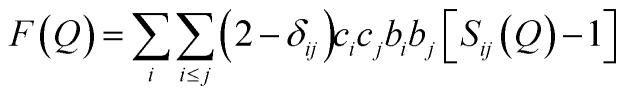
where *c*_*i*_ and *b*_*i*_ are the atomic fraction and neutron bound coherent scattering length of the *i*th atom type, respectively; and *δ*_*ij*_ is the Kronecker delta.

Only data arising from coherent, elastic scattering should be used to perform structural determination. Corrections for incoherent and inelastic scattering in addition to contributions from multiple scattering, sample container or self-scattering are corrected using the GudrunN and GudrunX packages for neutron and X-ray scattering data, respectively, which have been described in detail elsewhere.^29^

Neutron scattering measurements were performed using the Near and InterMediate Range Order Diffractometer (NIMROD) at the ISIS Neutron and Muon Source, which allows for the collection of scattering data across a wide *Q* range, from 0.02 to 50.0 Å^−1^.^30^ NIMROD is a total scattering instrument that is optimised for the study of liquids and other disordered materials across length scales that range from the atomic (less than 1 Å) to more than 30 nm. X-ray total scattering was performed using a Malvern Panalytical Empyrean diffractometer. The structure factor obtained from X-ray scattering provides better contrast from the inorganic ions than for a neutron-only scattering approach.

Isotopic substitution is employed in total scattering experiments to generate multiple total structure factors without significantly altering the structure of the system, in order to enhance the accuracy of the simulated structure. Specifically, in neutron scattering experiments of systems containing hydrogenous material, protium–deuterium substitution is commonly used owing to the differing neutron scattering lengths of the protium and deuterium isotopes of hydrogen (*b*_H_ = −3.74 fm and *b*_D_ = 6.67 fm).^31^ Here, we use the ideal case for a two-site substitution, *i.e.* H/D substitution of the water and TMG hydrogen atoms. This involves the seven substitutions outlined in Table 1, which allow the unique determination of the partial structure factors between the following three sets of atoms: the water hydrogen atoms, TMG hydrogen atoms and the remaining atomic sites in the system.^12^

**Table 1:** The seven isotopic substitutions used to study each of the two aqueous solutions containing TMG and either KCl or NaCl. These seven solutions are required for an ideal two-site substitution, where the TMG and water hydrogen atoms are substituted

Isotopic substitution	TMG	Water
1	H-TMG	H_2_O
2	H-TMG	D_2_O
3	HD-TMG	HDO
4	HD-TMG	D_2_O
5	D-TMG	HDO
6	D-TMG	H_2_O
7	D-TMG	D_2_O

### 2.2 Sample preparation

The aqueous solutions were prepared by weighing a known mass of either or both of H-TMG (Sigma-Aldrich, BioUltra, ≥99.0%) and D-TMG (ISIS Deuteration Facility); KCl (Thermo Scientific Chemicals, Puratronic™, 99.997%) or NaCl (Thermo Scientific Chemicals, Puratronic™, 99.998%); and ultrapure H_2_O (18.2 MΩ cm), D_2_O (Sigma-Aldrich, 99.9 atom% D), or HDO (a 1 : 1 molar mixture of H_2_O and D_2_O). Measurements were made at concentrations of 2 m TMG and 2 m salt, where m refers to the molal concentration defined by the moles of solute per kilogram of water, H_2_O. The molal concentration is only strictly defined for the solution containing only H_2_O as the solvent; solutions containing D_2_O were made at an equivalent molar ratio between all of the components in the solution containing only H_2_O.

We chose TMG as an archetypal zwitterionic osmolyte, alongside potassium and sodium chloride as typical cytosolic salts. These solutes and concentrations were carefully chosen as a compromise between ideal physiological conditions and experimental practicality: higher concentrations are required to obtain a good scattering signal from the solutes and the requirement to simulate a representative model of the system. Nevertheless, while the concentrations of the individual solutes are higher than those found in cytosol, they are found more widely in halophilic organisms, such as in the previously discussed examples.

For the neutron scattering measurements, the solution samples were transferred to flat, null scattering alloy cans (Ti_0.68_Zr_0.32_) of 1 mm thickness, and sealed with PTFE before being loaded onto a sample changer on the NIMROD instrument. For the X-ray scattering measurements, the solution samples were transferred to silica capillaries and sealed with beeswax.

### 2.3 Structural refinement

The Dissolve package was used to simulate and refine the structure of a model box of the two liquid solutions, at equivalent composition and densities (Table S1†) to the experimental solutions.^32^ Within Dissolve, the structure is initially simulated using a “reference” potential, details of which are discussed in the SI.^19,33–36^ Empirical Potential Structure Refinement (EPSR) is employed to refine the simulated structure towards the experimentally determined datasets. In this process, an additional “empirical” potential is derived from the difference between the experimental and simulated scattering data, which is subsequently applied to the simulation, resulting in simulated structures which are consistent with the experimental structure factors.

The refined simulations were used to perform subsequent analysis routines, including calculating radial and spatial distribution functions. Rather than performing these calculations over a specific snapshot of the refined simulation, they were performed over a trajectory of 10 000 frames. The resulting analysis therefore reflects a wide range of snapshots that are all valid solutions to the EPSR fitting process.

## 3 Results and discussion

**Fig. 1:**
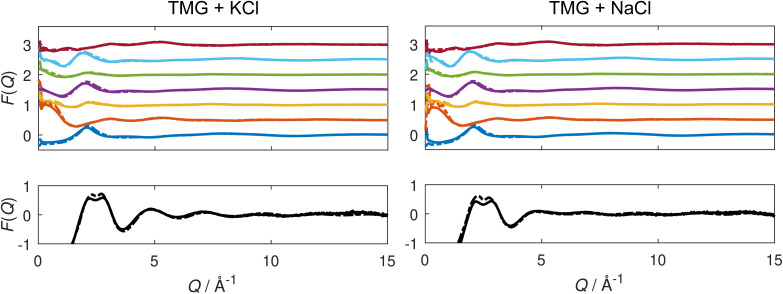
Measured (solid lines) and simulated (dashed lines) structure factors *F*(*Q*) for aqueous solutions containing TMG and KCl (left) or NaCl (right). Top: structure factors obtained from neutron diffraction in seven isotopic contrasts. Each dataset is vertically shifted by 0.5 for clarity, and the structure factors from isotopic substitutions 1–7 (Table 1) appear from bottom to top. 1 – dark blue; 2 – orange; 3 – yellow; 4 – purple; 5 – green; 6 – light blue; 7 – red. Bottom: measured (solid lines) and simulated (dashed lines) structure factor *F*(*Q*) obtained from X-ray diffraction.

The experimental and refined, simulated structure factors *F*(*Q*) for each of the contrasts are displayed in Fig. 1. Analogous figures for the total radial distribution functions *G*(*r*) are displayed in the ESI.† We note good agreement between experiments and simulations from the simultaneous fit across all neutron and X-ray measurements, such that the simulation is representative of the physical system. We now proceed to use the simulated system as the basis for our structural analysis.

### 3.1 TMG–ion interactions

**Fig. 2:**
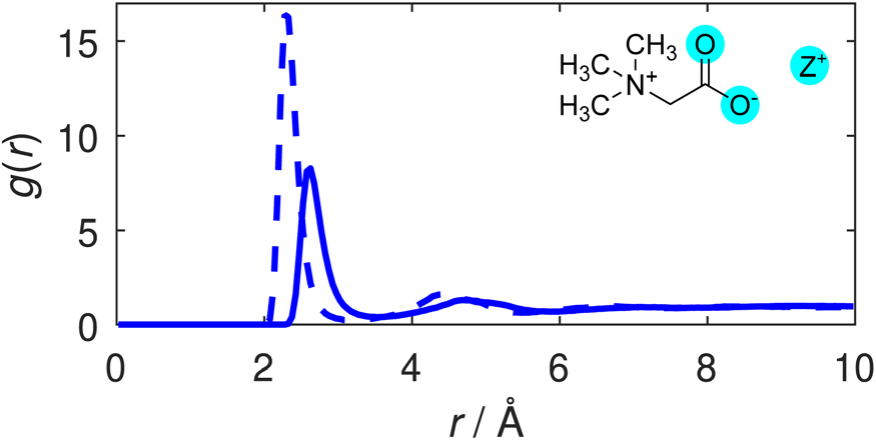
TMG oxygen-cation (O–Z) RDFs. The O–K (solid line) and O–Na (dashed line) distributions are shown.

We first consider the degree of coordination between TMG and the cationic species in solution. Radial distribution functions (RDFs) illustrating correlations between the TMG oxygen atoms (O) and the potassium or sodium cations (Z^+^) are displayed in Fig. 2. The sharp primary peak for both cations, located at 2.6 Å for the O–K RDF and 2.3 Å for the O–Na RDF illustrate a strong coordination between the TMG oxygen and the solution cations. These RDFs are largely reminiscent of the ion-water RDFs that can be found as part of a wider discussion of the ion hydration structure included in the ESI (Fig. S4†), showing an initial peak that is taller and at smaller distances in the O–Na RDF relative to the O–K RDF: a difference that can be easily attributed to differences in the ionic radii of the two cations. The relevant coordination numbers obtained from these TMG–ion RDFs are reported in Table 2. These, along with all coordination numbers, were calculated using eqn S2,† setting the limits of integration to zero for the lower limit and the radial cutoff for the upper limit, the distance at the first minimum in the relevant pair distribution function. The coordination number analysis reveals similar numbers for both cations; the taller peak in the O–Na RDF is therefore indicative of a tighter distribution for the coordination of Na^+^ around the TMG carboxylate group relative to the analogous K^+^ coordination.

**Table 2:** Comparison of the TMG oxygen–cation (O–Z) and water oxygen–cation (O_w_–Z) coordination numbers in both the KCl and NaCl-containing solutions

Environment	Coordination number	Cutoff *r*/Å
O–K	0.31	3.5
O–Na	0.34	3.2
K–O	0.54	3.5
Na–O	0.75	3.2
O_w_–K	0.20	3.5
O_w_–Na	0.18	3.2
K–O_w_	5.58	3.5
Na–O_w_	4.86	3.2

Whilst the TMG O–Z RDFs illustrate a well defined coordination of cations to the oxygen atoms, the coordination numbers of ∼0.3 for both cations appear to illustrate that this coordination is not strongly favoured. However, carboxylate oxygens are outnumbered by water oxygens by 14 to 1, which themselves have a coordination number of ∼0.2 for both cations – also displayed in Table 2 – thus demonstrating a preference for TMG–cation coordination compared to pure hydration.

We note greater distinction between the two cationic species by considering the Z–O coordination number. K^+^ has a CN of 0.54 with the TMG oxygen compared to 0.75 for Na^+^. Given the similarity between the O–Z coordination numbers for the two cationic species, this suggests either that the sodium ions are more likely to coordinate to both TMG oxygens at the same time (*i.e.* bidentate coordination) than potassium, or that they are more likely to be shared between two TMG molecules. The ion–oxygen coordination numbers are discussed in more detail in the ESI,† including running coordination numbers for the oxygen–ion interactions, *i.e.* as a function of radial distance (Fig. S6†).

**Fig. 3:**
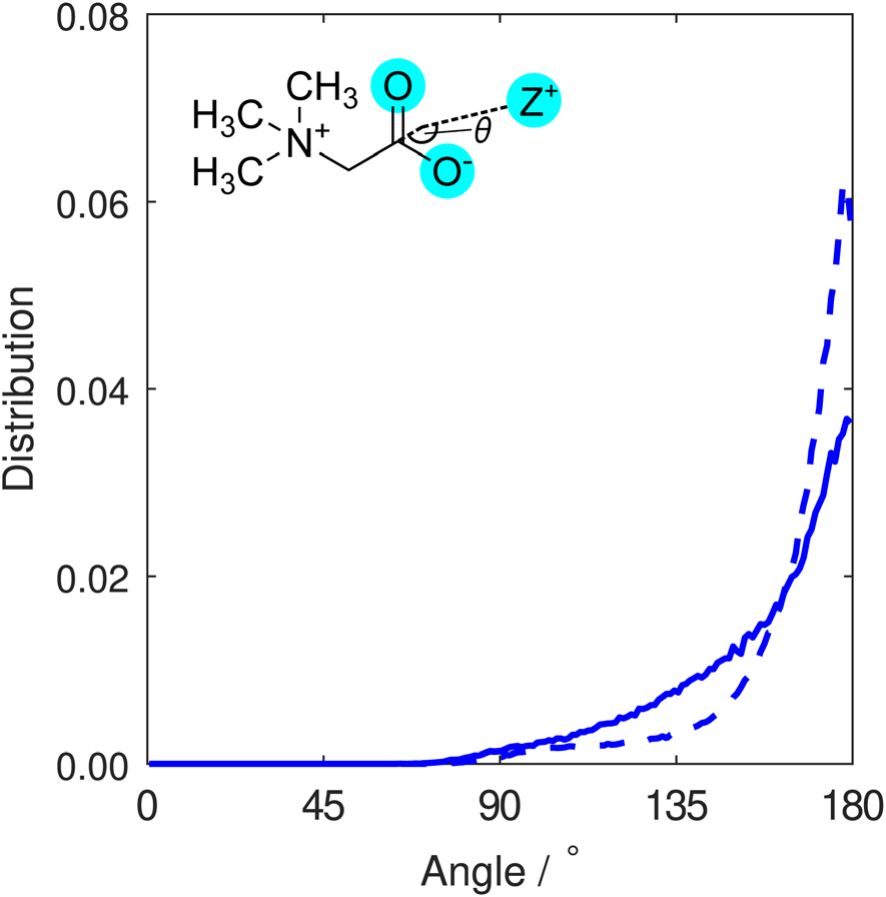
Distribution of the binding angles for potassium (solid line) or sodium (dashed line) cations coordinating to a TMG carboxylate group. This angle is defined as the angle between the vector connecting the cation and the centre of mass of the two carboxylate oxygens, and the vector connecting the same centre of mass and the carboxylate carbon atom (*i.e.* parallel to the dipole moment of the carboxylate group).

In order to differentiate between monodentate and bidentate coordination, in Fig. 3 we display the distribution for the angle formed between a vector directing from the cation to the centre of mass of the two carboxylate oxygen atoms, and another vector directing from this centre to the carboxylate carbon atom. This distribution was calculated for all cations found within distances from carboxylate oxygen atoms up to the first minimum in the O–Z RDFs (Fig. 2). Both K^+^ and Na^+^ ions are most likely to be located at binding angles of 180° (that is, equidistant to the two oxygen atoms), consistent with a bidentate binding geometry at the carboxylate group. We also note that the sodium ion distribution displays a tighter distribution about the modal angle relative to that of potassium. We interpret this to be indicative of sodium ions being bound more tightly to the carboxylate group and more often found in bidentate configurations than potassium ions, consistent with predictions from simulations,^28^ while the weak shoulder around 130° that is more pronounced for potassium is representative of some monodentate coordination.

### 3.2 TMG–TMG interactions and ion-mediated clusters

**Fig. 4:**
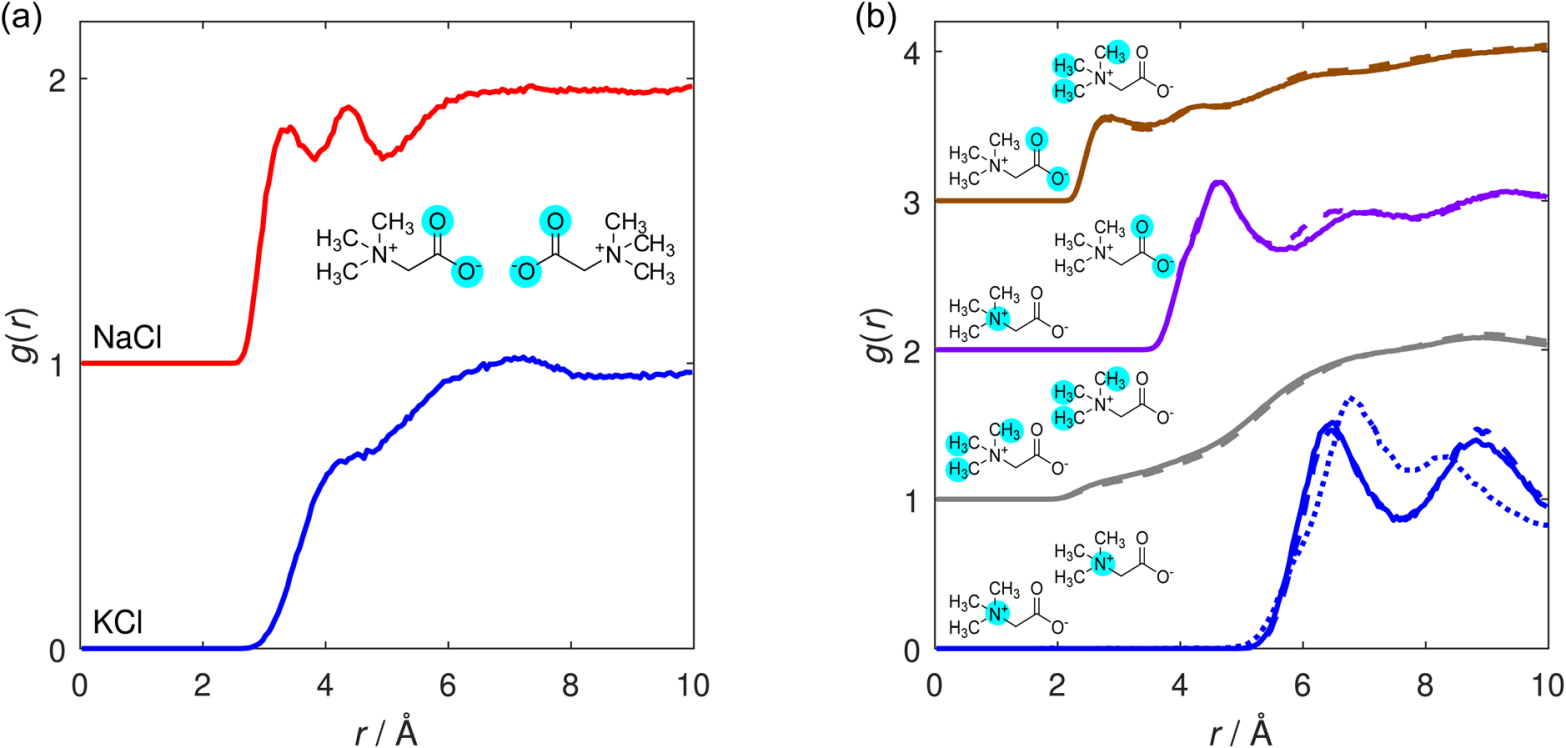
TMG–TMG RDFs calculated for a selection of intramolecular interaction sites. (a) Comparison of the O–O interactions in the presence of KCl (blue) and NaCl (red), vertically shifted for clarity. (b) N–N (blue), H–H (grey), N–O (purple) and O–H (brown) RDFs, calculated for the TMG + KCl (solid line) and TMG + NaCl (dashed line) solutions. H sites are considered on the trimethyl group only. For the N–N correlations, an additional dotted line for the case with no salt present is shown for comparison, from Gioacchino *et al.*^37^

We next consider interactions between TMG species themselves. The calculated TMG–TMG RDFs are displayed in Fig. 4. A comparison between the O–O RDFs in the presence of KCl and NaCl is shown in Fig. 4(a), and the remaining functions for a variety of interactions calculated from the two solutions are shown in Fig. 4(b).

The O–O RDF in the NaCl reveals two distinct peaks at distances of 3.4 Å and 4.4 Å, in contrast to the single shoulder at a distance of 4.3 Å when KCl is present. This latter case is more similar to results from a previous study by Gioacchino *et al.* determining the structure of a TMG-containing aqueous solution with no salt present.^37^ In this work, a single peak at 4.1 Å was reported in the O–O RDF, attributed to water-mediated association of TMG molecules *via* their carboxylate groups. In the presence of NaCl, we attribute the O–O correlation peak at smaller distances to the formation of ion-mediated interactions between TMG molecules; the tight-binding between the ions and the TMG carboxylate group reduces the distance between carboxylate oxygen atoms on different molecules, relative to the case with no salt. We infer from the presence of the double peak in the presence of NaCl relative to KCl that the Na-mediated association of TMG is more ordered and tightly bound than the K-analogue. This is supported by Fig. 3, which indicates similar sodium localisation.

In the context of understanding TMG–TMG associations, it is also worth considering the N–N RDF shown in Fig. 4(b), which displays two distinct peaks at 6.5 Å and 8.8 Å. These results are qualitatively similar to the double peak reported for the case with no salt,^37^ where the peak at a smaller distance is attributed to direct TMG–TMG chain configurations driven by electrostatic interactions due to opposite charges on the N and O sites, owing to its zwitterionic character (*i.e.* head-to-tail clustering). The peak at the larger distance was attributed to the presence of water-mediated association of TMG molecules, as we discussed previously; which, in our case, now includes ion-mediated associations. It is interesting to note in the N–N RDFs that the second of these peaks is significantly smaller in height than the first with no salt present, whereas we observe a peak much more similar in height from our results in the presence of KCl or NaCl. We can obtain a measure of this by comparing the coordination number of each peak. For the case with no salt^37^ the coordination number of the second peak is 2× larger than for the first, while for KCl it is 2.6× larger and for NaCl 2.9× larger (although we emphasise that in all cases similar N–N distances can be obtained through other means as well, *e.g.* from molecules located side-to-side rather than end-to-end, so are not indicative of the absolute ratio of head-to-head *versus* head-to-tail interactions). From these results we infer a significantly increased likelihood of TMG–TMG association *via* an ion-mediated carboxylate interaction rather than a head-to-tail chain interaction driven by the TMG zwitterionic character.

**Fig. 5:**
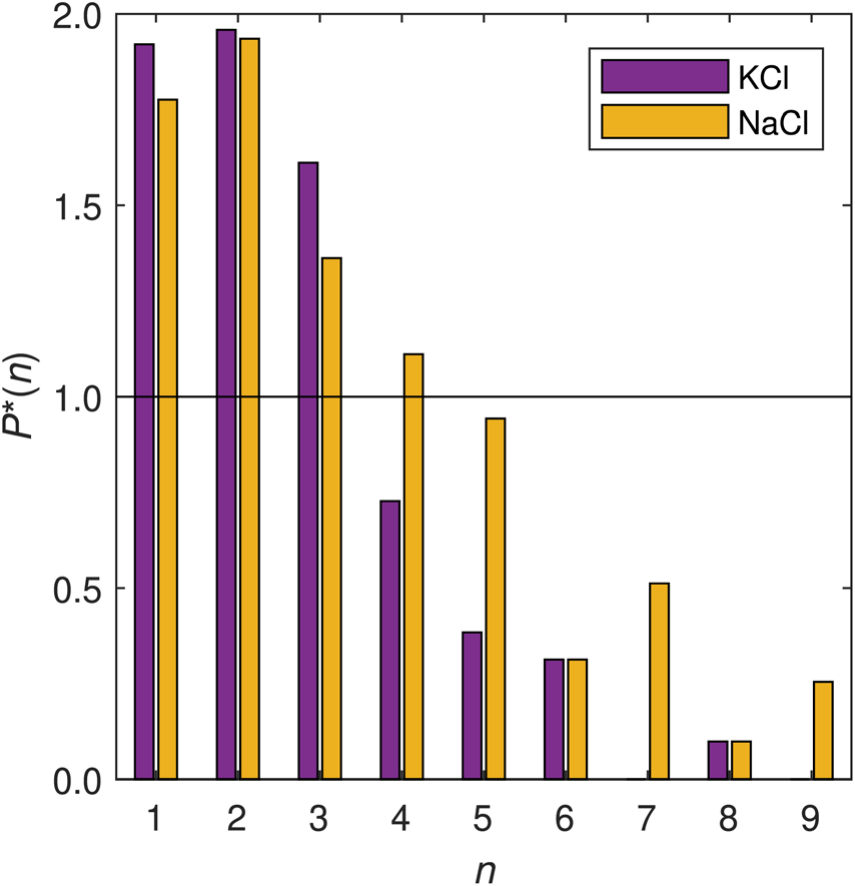
Normalised distribution function for finding cluster of size *n*, calculated by considering O–O interactions with a radial cutoff at 4.9 Å. The reported probability P*(*n*) is the calculated probability of finding a cluster of size *n* in the box, scaled by the percolation threshold *P*_p_(*n*). The threshold is given by *P*_p_ = *αn*^−1.2^ where the exponent arises from the universal Fisher exponent, calculated for a three-dimensional system of non-interaction spheres and which is independent of any lattice geometry^39,40^ and *α* is a normalisation factor.

Next, we further investigate these inferences *via* a cluster analysis, quantifying the size of TMG clusters where the TMG oxygen to oxygen distance cutoff is set at 4.9 Å, obtained from the second minimum in the O–O (NaCl) RDF in Fig. 4(a). Here, we employ a method used previously to study clustering and percolation in a number of experimental systems, including ionic liquids,^38^ calculating the probability *P*(*n*) of finding a molecules of a specific type within a cluster of size *n*. In Fig. 5 we show this cluster analysis for TMG–ion clusters in KCl and NaCl-containing solutions, where the reported probability is scaled by the calculated percolation threshold P*(*n*) = *P*/*P*_p_, in order to ensure that the identified clusters are statistically significant, and not due to random fluctuations.

The analysis reveals a TMG population dominated by monomers (individual TMG molecules), followed by dimers (water or ion-bridged TMG pairs, such as the example shown in Fig. S3†) and finally trimers. Unlike the differences we observe in the O–O RDFs, there is no significant difference in the extent of association across the two salt solutions. Other clusters of size *n* ≥ 4 were found in both salt solutions, typically with greater frequency in NaCl, suggesting more extensive large scale structure than for KCl. However such structures were only statistically significant for *n* = 4 – that is, larger assemblies were also detected but with probabilities lower than the percolation threshold and thus no more likely than might be expected from random fluctuations.

Therefore, even though the structure of such associates is seen to vary with ion identity, *i.e.* more ordered TMG–ion and TMG–TMG interactions in the presence of sodium, the size of the associates does not change dramatically. This is supported by Fig. 4(b), which shows similar second peak heights in the N–N RDF, indicating similar coordination numbers. However, we emphasise that neither the cluster analysis nor the RDF strictly captures the shape or stability of any aggregates. It may be that one ion species creates more long-lived, more compact or more ordered aggregates. It also does not distinguish between head-to-head configurations brought about by ion-bridging and those brought about by water-bridging, which are still likely to occur.

**Fig. 6:**
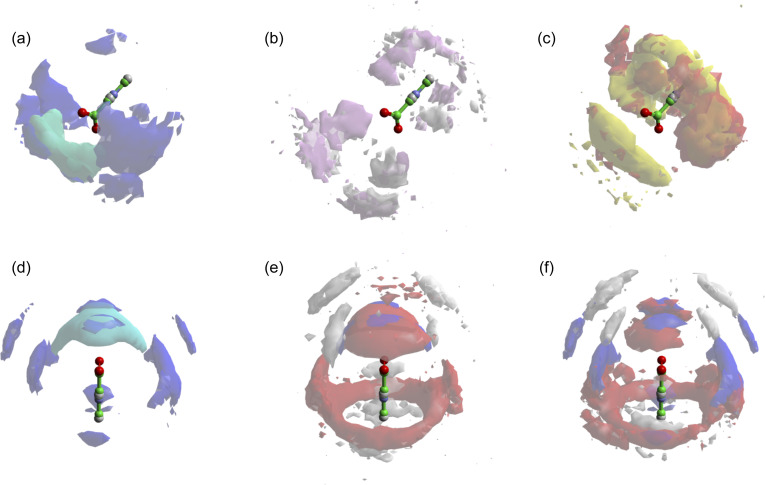
TMG carboxylate – TMG carboxylate SDFs, showing most likely locations for: (a) all orientations for both NaCl (light blue) and KCl (dark blue); (b) parallel orientation for NaCl (pink) and KCl (white); (c) antiparallel orientation for NaCl (yellow) and KCl (red); (d) same as (a) but different orientation; (e) NaCl, for all orientations (blue), antiparallel (red) and parallel (white); (f) KCl for all orientations (blue), antiparallel (red) and parallel (white). Isosurfaces are drawn of the 10% most likely configurations of carboxylate centres-of-mass.

To establish the organisation of any TMG clusters, we instead turn to the spatial density function (SDF). A comparison of SDFs calculated for specific TMG–TMG orientations supports the idea that the zwitterion-ion clusters are more compact in the presence of sodium than for potassium. The most likely location for a TMG molecule next to another TMG molecule is highly localised at the carboxylate group for sodium chloride, but is much less localised for potassium chloride, forming off-axis diffuse lobes either side of the carboxylate group in addition to a weaker on-axis lobe (Fig. 6(a) and (b)). By only considering TMG molecules oriented parallel or anti-parallel to each other, we can draw a further distinction between the two cationic species. For sodium, the SDF maxima is also the antiparallel SDF (*i.e.* head-to-head) maxima, and there is also a ring around the quaternary ammonium, suggesting side to side head-to-tail localisation (Fig. 6(c) and (e)). For potassium, the anti-parallel SDF is similar in character to the sodium, but the head-to-tail ring is broader (Fig. 6(c) and (f)). One might expect the cationic species to play no role in the head-to-tail interaction, but in fact for the potassium ion this ring is at larger distances (Fig. 6(c)). This could be due to presence of off-axis head-to-head TMG at the carboxylate group for potassium *versus* on-axis head-to-head TMG for sodium, resulting in a different overall coordination structure. On the other hand, we see little difference between the parallel SDF between the two cationic species (Fig. 6(b), (e) and (f)). In both cases the most likely locations are off-axis, whether at the carboxylate or quaternary ammonium group, less well defined than the anti-parallel SDFs and at very similar distances. This supports the idea that the cationic species plays little role in head-to-tail chain formation, which does not need to be propagated *via* an intermediate species. This analysis also emphasises the clear role of cationic species in forming head-to-head clusters: if these interactions were primarily mediated by water molecules, we would expect little difference in the SDFs between the two salt species.

Our experimental results thus confirm the presence of TMG–ion associates in solution, giving credence to the idea that TMG molecules can retain inorganic ions in close proximity, potentially imparting protection from the salting out of proteins or biomolecules.

### 3.3 TMG hydration structure

We conclude by considering the hydration structure of the TMG molecules. The unique hydration structure of TMG resulting from its antagonistic dipolar and hydrophobic properties is thought to contribute to its stabilising effect on biomolecules.^41^ We consider correlations between TMG and surrounding water molecules, centred on two contrasting sites on the osmolyte molecule: first, the anionic, carboxylate group, where correlations are calculated from the carboxylate oxygen atoms (O); and second, the cationic, quaternary ammonium group, where correlations are calculated from the methyl carbon atoms (C_m_).

**Fig. 7:**
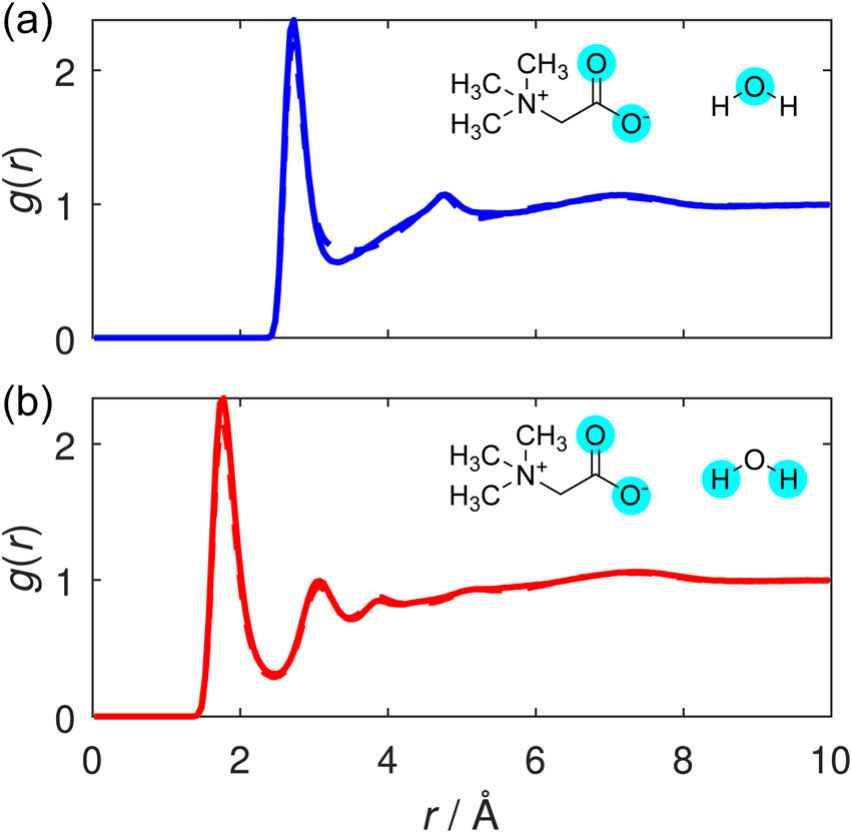
(a) O–O_w_ and (b) O–H_w_ RDFs, shown for the TMG + KCl (solid line) and TMG + NaCl (dashed line) solutions. (Note, the solid and dashed line are almost exactly on top of each other for both panels.)

Considering first the carboxylate–water interactions, the O–O_w_ and O–H_w_ RDFs are displayed in Fig. 7(a) and (b), respectively, and the corresponding hydration coordination numbers calculated from these are displayed in Table S5.† As expected from a negatively charged, hydrophilic carboxylate group, our RDFs illustrate a strong hydrogen bonding environment, with a well-defined, sharp peak in both. The primary peak in the O–H_w_ peak is located at 1.8 Å, the hydrogen bond distance between TMG and water. This shorter hydrogen bond suggests that the hydrogen bond interaction between TMG and water is potentially slightly stronger than the hydrogen bonds between water molecules themselves in this solution. This increase in strength can be attributed to the negatively charged carboxylate oxygens, and has been observed in previous neutron scattering structural studies of aqueous solutions containing osmolytes including both TMAO or TMG.^22,37,42^

**Fig. 8:**
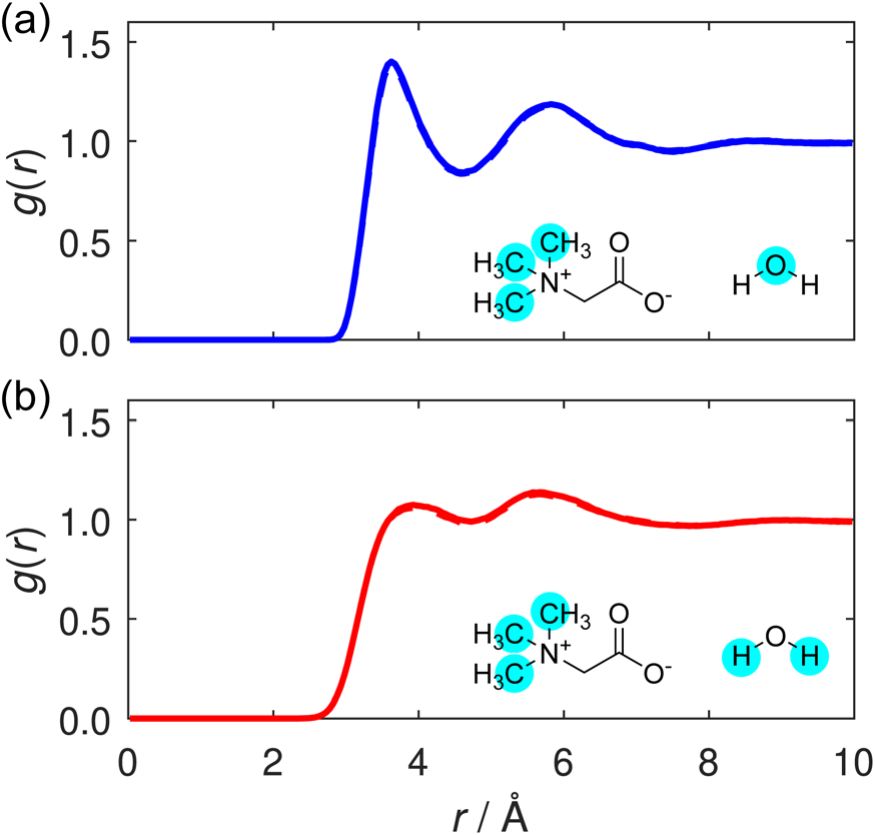
(a) C_m_–O_w_ and (b) C_m_–H_w_ RDFs, shown for the TMG + KCl (solid line) and TMG + NaCl (dashed line) solutions. (Note, the solid and dashed line are almost exactly on top of each other for both panels.)

Next, we consider the trimethyl ammonium–water interactions, and the calculated RDFs are displayed in C_m_–O_w_ and C_m_–H_w_ RDFs are displayed in Fig. 8(a) and (b), respectively. These RDFs display clear peaks, although the primary peaks – located at 3.6 Å for C_m_–O_w_ and 3.9 Å for C_m_–H_w_ – are notably broader than those in the carboxylate–water RDFs. Although the two RDFs are more qualitatively similar than those presented for the carboxylate oxygen analogues, the slight difference in the primary peak position implies a net favourable orientation of water molecules to point their oxygen atoms towards the methyl group. This observation suggests that the water molecules are somewhat oriented due to the presence of the positive charge on the TMG nitrogen atom, and as such the interaction between the TMG quaternary ammonium group and surrounding water molecules is not purely hydrophobic at this moiety. Similar observations have been made previously in molecular dynamics simulations of aqueous TMG solutions.^41,43^ This difference implies that the water molecules surrounding the carboxylate group are more strongly ordered than those surrounding the methyl groups: something that is to be expected between more hydrophilic or hydrophobic regions of an osmolyte molecule.

**Fig. 9:**
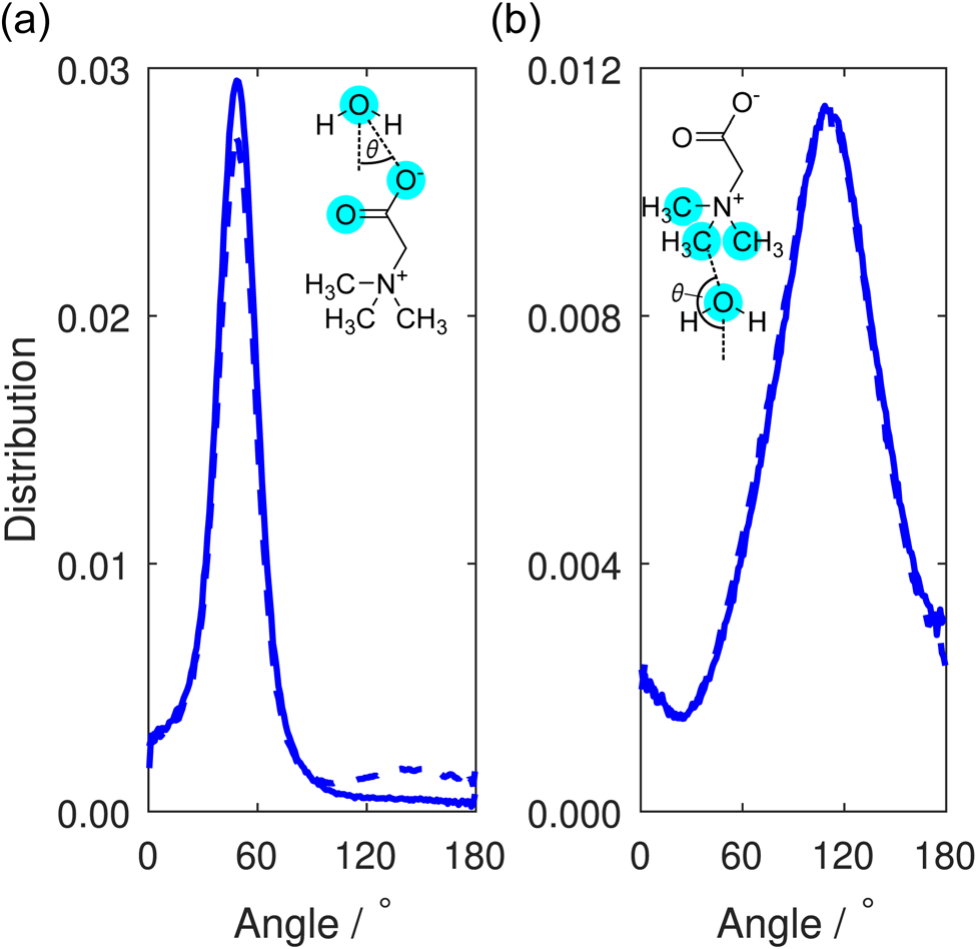
Distribution of the dipole angles for water molecules hydrating (a) the TMG carboxylate group or (b) TMG methyl group. This angle is defined as the angle between the vector connecting (a) a carboxylate oxygen or (b) methyl carbon and the hydrating water molecule, and the vector corresponding to the dipole moment of the water molecule. Distributions are shown for the TMG + KCl (solid line) and TMG + NaCl (dashed line) solutions.

This orientational geometry can be further interrogated by calculating the angular distribution of the water dipole for the hydrating water molecules at the two sites. In these distributions, displayed in Fig. 9, the angle is calculated between the vector directing along the water dipole moment and the vector connecting the water oxygen to either the carboxylate oxygen or the methyl carbon on the TMG molecule. Only water molecules located at distances smaller than the first minimum in the O–O_w_ RDF at 3.3 Å (Fig. 7) contribute to the distribution.

In Fig. 9(a), the distribution contains a single sharp maximum located at 47.5°, characteristic of the hydrogen bond-accepting properties we inferred from the TMG–H_2_O RDFs (Fig. 7 and 8) and that we might expect for a negatively charged, hydrophilic moiety. This peak is indicative of a hydrating water molecule geometry whereby it is pointing one of its hydrogen atoms towards the carboxylate oxygen. It is worth comparing these results to the analogous distribution for the water–water interactions, displayed as part of a wider discussion of the bulk water structure in the ESI.† This distribution (Fig. S8†) contains two peaks rather than the single peak seen here. This difference can simply be ascribed to the water molecules acting as both hydrogen bond-donators and acceptors, whereas the TMG carboxylate group can only accept hydrogen bonds. However, we also note a small peak at ∼140° for the NaCl system, which is not the case for KCl. We can attribute this to highly localised sodium (see Fig. 3), in effect, fulfilling the role of a pseudo-cationic group for the TMG molecule which can in turn coordinate with the water oxygen atom. It is likely that potassium fulfils a similar function, but due to being much less localised, this is not visible in the dipolar angle distribution.

Turning to the water dipole angle distribution around the methyl group in Fig. 9(b), the distribution again contains a single peak, but it is instead located at an angle of 109.5° and is much broader. As we inferred from the TMG–H_2_O RDFs, despite the more hydrophobic nature of the methyl groups there is still some ordering of the hydrating water molecules around this region of the TMG molecules owing to the positive charge on the nitrogen atom. This angular distribution is consistent with this picture as it suggests that the water molecules orient their hydrogen atoms away from these methyl groups, instead orienting their oxygen electron density towards to the positively charged region of the zwitterion. The increased width of the distribution relative to the carboxylate analogue can be easily explained through the fact that this functional group would not be expected to hydrogen bond with the surrounding water molecules, and as such there is much more disorder present in this angular distribution.

The RDFs presented in this section are largely similar in nature when compared to the literature results with no salt present. It is clear, therefore, that the presence of salt in the solution does not have significant implications for the hydration of TMG, and the osmolyte is able to retain its hydration shell even at high concentration, in agreement with predictions from simulation.^28^

## 4 Conclusions

In this work, we have used a total scattering approach to determine the structure of mixed osmolyte–ion solutions, revealing molecular-level details including osmolyte–ion interactions and clustering. Specifically, we study solutions containing the common osmoprotectant, trimethylglycine (TMG), in the presence of either KCl or NaCl as a co-solute. We employ H/D isotopic substitution to generate multiple neutron scattering contrasts in addition to a further X-ray contrast, and perform structural determination with an Empirical Potential Structure Refinement (EPSR) approach using the Dissolve package. We reveal some interesting details concerning the nature of TMG–cation interactions, observing that the carboxylate group exhibits clear binding to the cations in the solution, promoting strong head-to-head TMG–TMG interactions *via* bridging ions. This contrasts to the case with no salt, where head-to-tail dipolar chain interactions dominate, with weaker head-to-head interactions also present, mediated by hydrogen-bonded water. Sodium ions appear more highly localised than potassium ions, both in terms of distance to the carboxylate oxygen atoms and the distribution of angles with which they bind, owing to the difference in ion size and preference for bidentate coordination. This difference also enables more structurally ordered TMG–TMG associates in the presence of sodium ions, observed from the clear structuring in the TMG O–O radial distribution function, in the TMG carboxylate–water dipolar angle and most significantly in the TMG–TMG SDFs.

The observation of osmolyte–ion binding has implications for the role of TMG as an osmoprotectant in the cellular environment, providing mechanistic detail for the protection of proteins and other biomolecules from an excess of salt in the cytosolic fluid, and the bioavailability of these metabolically valuable molecules. Larger, more stable aggregates might help suppress the harmful effect of osmotic stress, at the expense of withholding them from metabolic processes. Taken together, we observe that the TMG–TMG coordination and the hydration of the TMG carboxylate group are not only influenced by the addition of salt, but show strong cation species dependence. This work demonstrates the complex relationships that dictate self-assembly and solvation structure in cytosol solutions, and highlights the role of zwitterionic osmolytes in ion-containing solutions alongside their other osmoprotection mechanisms, such as their ability to maintain water structure at high pressure, and their capacity to interact with, and modulate interactions between, biological surfaces.

## Data availability

Raw neutron scattering data for this article are available in the ISIS Data Catalogue (https://doi.org/10.5286/ISIS.E.RB2220174-1). Dissolve simulations, GudrunN and GudrunX (including raw X-ray scattering data) files are available from the Oxford University Research Archive (https://doi.org/10.5287/ora-deg26axdb).

## Author contributions

Conceptualisation – K. J. A., J. E. H., S. P., O. L. G. A., T. F. H., T. L. H., G. N. S. Investigation – K. J. A., T. S. G., S. M., Y. K. C. F., O. L. G. A., T. F. H. Formal analysis – K. J. A., S. M., J. E. H. Resources – J. P. T., Y. C. Supervision – S. P., J. E. H. Writing, original draft – K. J. A., J. E. H. Writing, review and editing – all authors.

## Conflicts of interest

There are no conflicts to declare.

## Supplementary Material

SC-OLF-D5SC00286A-s001
