# Supplementary material for: Specific ion effects enhance local structure in zwitterionic osmolyte solutions
Source: Chem Sci. 2025 Mar 17;16(16):6770–9. doi: 10.1039/d5sc00286a (PMC11934062; doi:10.1039/d5sc00286a)
Supplement: SC-016-D5SC00286A-s001 [file SC-016-D5SC00286A-s001.pdf]

Supplementary Information (SI) for Chemical Science.
This journal is © The Royal Society of Chemistry 2025

Supplementary Information

Specific ion effects enhance local structure in zwitterionic osmolyte solutions

Kieran J. Agg[a], Timothy S. Groves[a], Shurui Miao[a], Y. K. Catherine Fung[a], Oliver L. G. Alderman[b], Thomas F. Headen[b], Terri-Louise Hughes[b], Gregory N. Smith[b], Tristan G. A. Youngs[b], James P. Tellam[b], Yao Chen[b], Susan Perkin[a] and James E. Hallett*[c]

[a] *Physical and Theoretical Chemistry Laboratory, Department of Chemistry, University of Oxford, Oxford OX1 3QZ, UK.*

[b] *ISIS Neutron and Muon Source, Rutherford Appleton Laboratory, Didcot OX11 0QX, UK.*

[c] *Department of Chemistry, School of Chemistry, Food and Pharmacy, University of Reading, Reading RG6 6AD, UK. E-mail: j.e.hallett@reading.ac.uk.*

Materials and methods: further details

Solution densities

The density of the two hydrogenous liquid samples investigated using total scattering were measured using a density meter (Anton Paar DMA 4100 M). A small amount of the sample ($\sim$ 1 mL) is inserted into a U-shaped tube which is excited into an oscillation. The frequency of this oscillation is determined and analysed, from which the density of the sample can be calculated to a precision of 0.0001 g cm^{-3}. The measured mass and calculated atomic number densities are reported in Table S1.

Table S1. Measured mass and calculated atomic number densities of the two aqueous solutions containing TMG and either KCl or NaCl.

Solution	ρ / g cm^{-3}	ρ / atoms Å^{-3}
2 m H-TMG + 2 m KCl (H_2O)	1.0978	0.09967
2 m H-TMG + 2 m NaCl (H_2O)	1.0897	0.10129

Reference potential details

For each of the two studied solutions, a model system was generated using the Dissolve package containing 9330 water molecules and 340 of each of the solute species (TMG, K^+ or Na^+, and Cl^-), in a cubic box of side length 70.3 Å. This box size was deemed appropriate as a compromise between, enhanced statistics on the one hand, and on the other, greater computing power required for larger box sizes. When generating a simulation box for this purpose, an additional consideration is the limit on the length scale over which structural correlations can be calculated. The method used in this work places a limit on the maximum length scale at half the box side length; the simulated box is of a large enough size to investigate all of the structural correlations of interest.

The simulation requires a classical molecular dynamics forcefield to be applied to each of the species in the simulation. For the water molecules in our simulation, we use the flexible single point-charge water model (SPC/E)[1]. For the TMG molecules, we extract a forcefield using the LigParGen tool, which provides bond, angle, dihedral and Lennard-Jones OPLS-AA parameters, as well as partial atomic charges[2–4].

The forcefield for the K^+, Na^+ and Cl^- species used in the Dissolve simulations of our two model cytosol solutions was generated using Eq. S1 for the simplest intermolecular potential - comprising the sum of a Lennard-Jones and a Coulomb term. The parameters for this potential were obtained from literature neutron diffraction studies on aqueous solutions of KCl and NaCl, which are displayed in Table S2[5].

$$U_{\text{inter}} = 4\epsilon_{ij}\left[\left(\frac{\sigma_{ij}}{r_{ij}}\right)^{12} - \left(\frac{\sigma_{ij}}{r_{ij}}\right)^{6}\right] + \frac{q_i q_j}{4\pi\epsilon_0 r_{ij}} \tag{S1}$$

Table S2. Lennard-Jones and Coulomb parameters for the K^+, Na^+ and Cl^- species in the Dissolve simulations.

Species	ϵ / kJ mol^{-1}	σ / Å	q / e
K	0.5144	2.94	1
Na	0.5144	2.29	1
Cl	0.566	4.191	-1

Calculation of coordination numbers

Coordination numbers were calculated using the Dissolve package, by spherically integrating $g_{\alpha\beta}(r)$ between the limits r_1 and r_2, as described by Eq. S2:

$$N_{\alpha}^{\beta} = \rho \int_{r_1}^{r_2} 4\pi r^2 g_{\alpha\beta}(r) dr \tag{S2}$$

where N_{α}^{β} is the coordination number of atom β around a central atom α, ρ is the atomic number density of the sample and $g_{\alpha\beta}$ is the radial distribution function (RDF). In all analysis performed in this work, the lower limit of integration r_1 was set to zero and the upper limit r_2 was set to the radial cutoff, the distance at the first minimum in the relevant pair distribution function, as required to perform the calculation across the first coordination shell.

In the coordination number analysis performed in this work, the error in the mean is small owing to the large number of simulation snapshots that contribute to its calculation. This error is approximately 0.01 for all coordination numbers; all values are reported to two decimal places to reflect this accuracy. Additionally, for most coordination number distributions, it is also possible to determine their standard deviations, a measure of the width of the distribution. These are reported alongside the mean coordination number where appropriate. However, for coordination numbers smaller than 1 it is not meaningful to calculate a standard deviation as they are not symmetrical distributions, and they are not reported in these cases.

Total radial distribution functions

Fig. S1 show the experimentally measured (solid line) and simulated (dotted line) total radial distribution functions $G(r)$ factors for all of the seven neutron isotopic contrasts, in addition to that determined by X-ray diffraction, measured for the TMG + KCl solution.

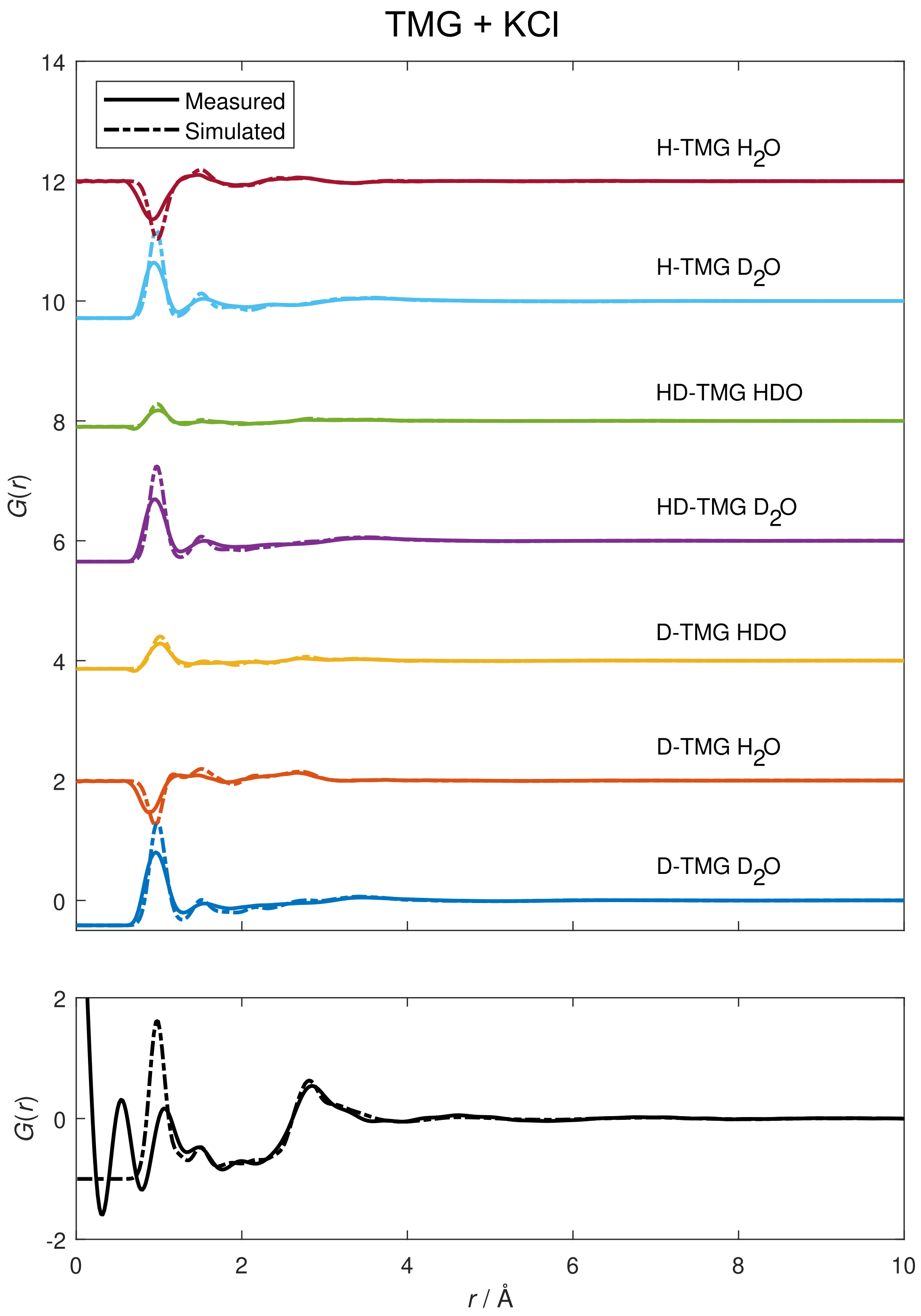

Figure S1. Measured (solid lines) and simulated (dashed lines) total radial distribution functions $G(r)$ for aqueous solutions containing TMG and KCl. Top: Total radial distribution functions obtained from neutron diffraction in seven isotopic contrasts; each dataset is vertically shifted for clarity. Bottom: Total radial distribution function $G(r)$ obtained from X-ray diffraction.

The analogous total radial distribution functions $G(r)$ for the NaCl-containing system are shown in Fig. S2.

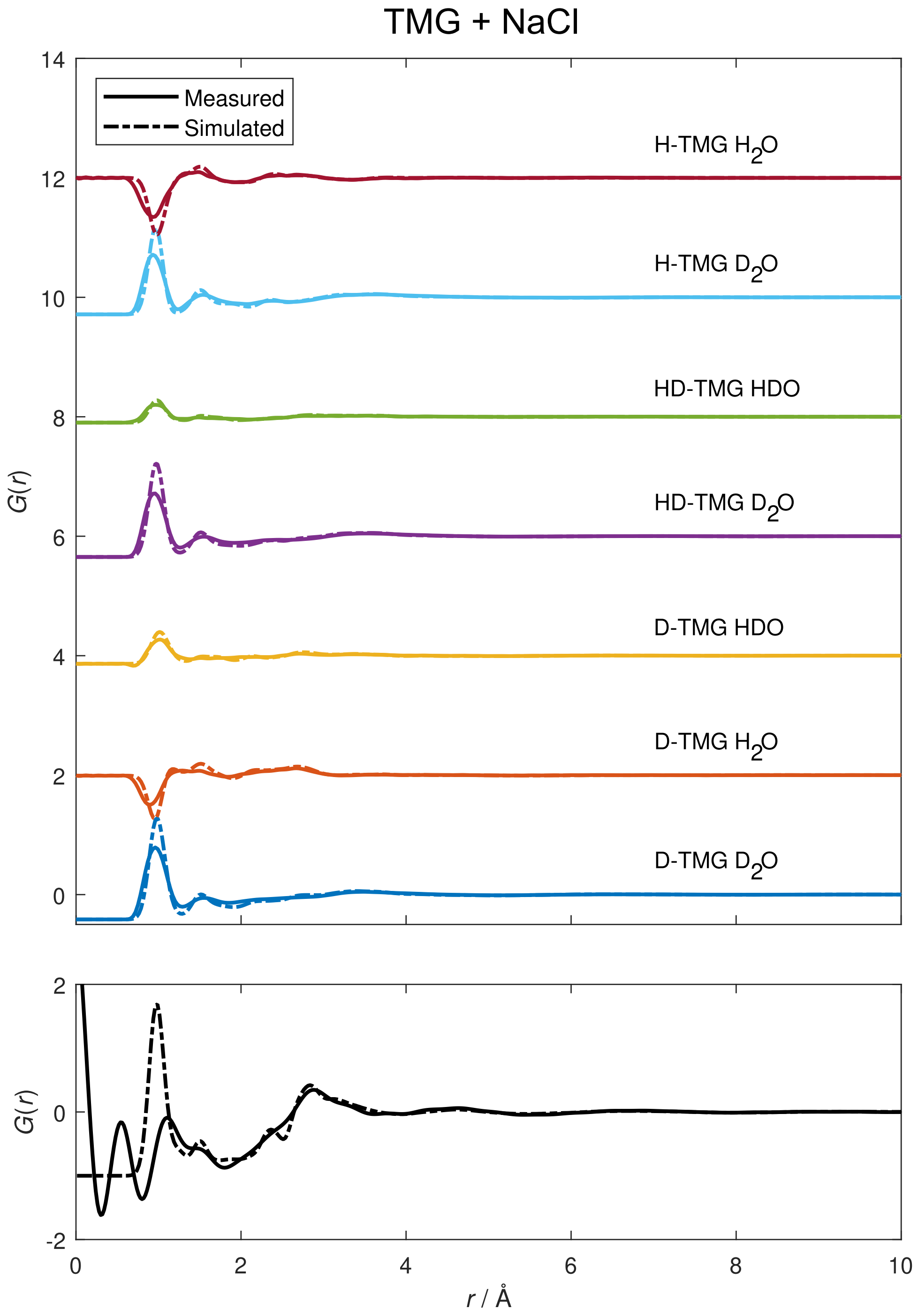

Figure S2. Measured (solid lines) and simulated (dashed lines) total radial distribution functions $G(r)$ for aqueous solutions containing TMG and NaCl. Top: Total radial distribution functions obtained from neutron diffraction in seven isotopic contrasts; each dataset is vertically shifted for clarity. Bottom: Total radial distribution function $G(r)$ obtained from X-ray diffraction.

Ion-mediated TMG clusters

Fig. S3 shows an example of an ion-mediated TMG dimer present in the simulated structure, as found in the cluster analysis (Fig. 5).

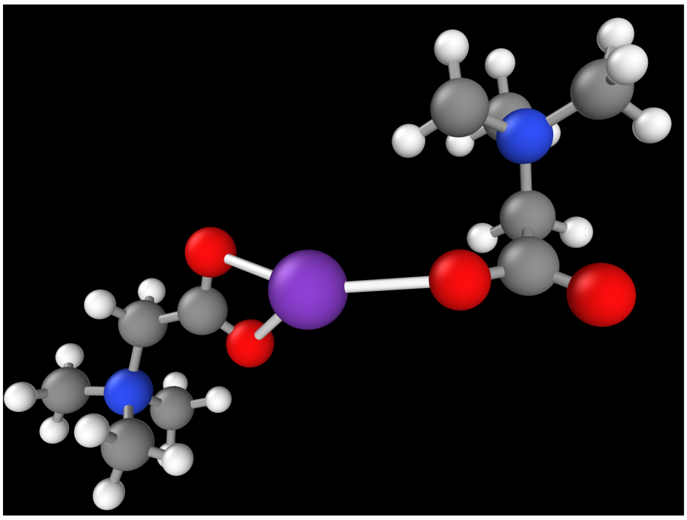

Figure S3. An ion-mediated TMG dimer found in the simulated structure. For emphasis, bonds are shown between the O and K atoms. Atom types: C - grey; H - white; O - red; N - blue; K - purple.

Ion hydration structure

In this section, we consider the hydration structure of the monovalent ions in our solutions, namely K^+ or Na^+ and Cl^- ions. There is a growing body of evidence, from a variety of spectroscopic, scattering and simulation studies, that simple monovalent ions only perturb the structure of water significantly within their first hydration shells[6,7].

We will begin this aspect of the structural interrogation by considering the RDFs calculated between the cation Z^+ (either K^+ or Na^+) and the water O_w and H_w atoms, displayed in Fig. S4 (a); and equivalently for the Cl^- anion in both solutions, displayed in Fig. S4 (b).

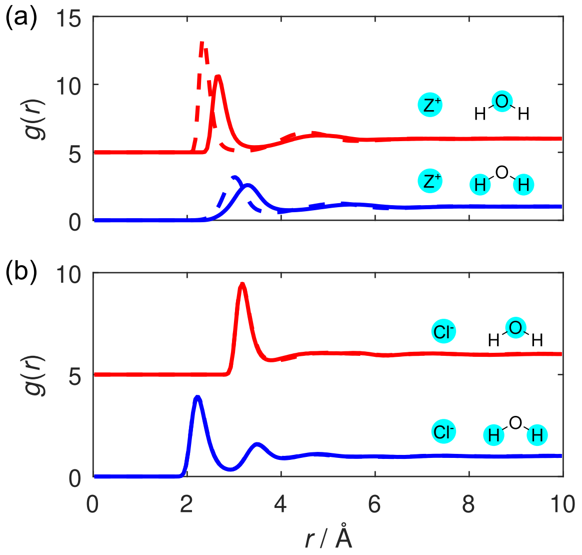

Figure S4. (a) Cation-water and (b) anion-water RDFs, shown for: $Z^+ = K^+$ (solid line) and $Z^+ = Na^+$ (dashed line). The RDFs calculated between the ions and O_w are shown in red and H_w in blue; these functions have been shifted for clarity.

The ion-water RDFs are consistent with the results obtained from structural studies upon aqueous solutions containing only monovalent ions from Mancinelli *et al.*[5] The position of the primary peak in the K-O_w RDF can be found at 2.7 Å, and at 2.3 Å in the Na-O_w RDF. Similarly, the position of the primary peak in the Cl-H_w RDF can be found at 2.2 Å. In their work, Mancinelli *et al.* observe that the primary peak position of these RDFs is insensitive to the ion concentration, even if the peak intensity and water coordination number is indeed sensitive to concentration. Our results are consistent with this, and demonstrate that these peak positions are also insensitive to additional osmolyte co-solutes present in the solution. From Fig. S4 (b), it is clear that the hydration structure of the chloride anion is unaffected by the identity of the cation.

All ion-water coordination numbers are displayed in Tables S3 and S4 for KCl and NaCl respectively, and satisfactorily reproduce the weakly concentration sensitive results reported by Mancinelli *et al.*[5].

Further analysis of the ion hydration structures can be performed by calculating the water dipole angle distribution of molecules hydrating a central ion. This distribution was calculated for all ion species - K^+,

Table S3. Comparison of the ion-water coordination numbers in the KCl-containing solutions.The standard deviation of the distributions are displayed in parentheses.

Environment	Coordination number	Cutoff r / Å
K-O_w	5.58 (1.26)	3.5
K-H_w	16.04 (2.34)	4.1
Cl-O_w	6.59 (1.16)	3.8
Cl-H_w	6.08 (1.19)	2.9

Table S4. Comparison of the ion-water coordination numbers in the NaCl-containing solutions. The standard deviation of the distributions are displayed in parentheses.

Environment	Coordination number	Cutoff r / Å
Na-O_w	4.86 (1.21)	3.2
Na-H_w	12.34 (2.31)	3.7
Cl-O_w	6.87 (1.04)	3.8
Cl-H_w	6.25 (1.11)	2.9

Na^+ and Cl^- - by considering all of the hydrating water molecules in the first coordination shell of each ion. The results for the potassium and sodium cations are displayed in Fig. S5 (a), and those for the chloride anions in Fig. S5 (b). Comparing the distribution for the two cations, we find a tighter distribution in the case of Na relative to that of K, implying a more disordered hydration structure around the K cation than for Na. This is akin to the findings in the main text from the carboxyl-ion binding angle distribution (Fig. 3).

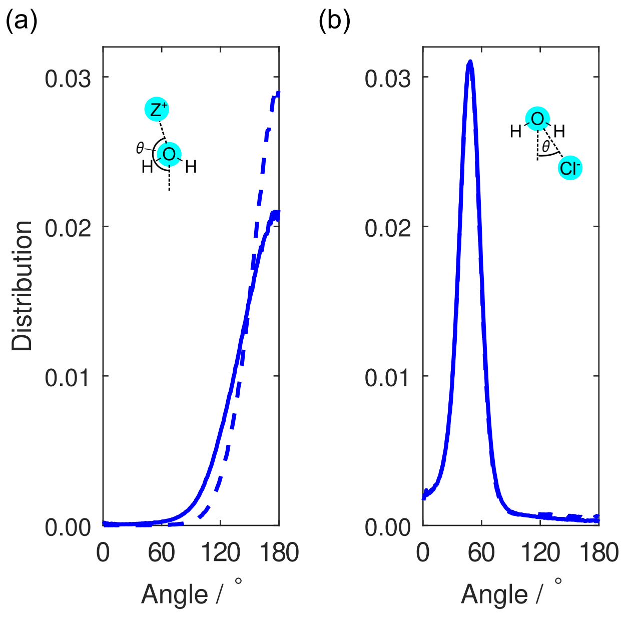

Figure S5. Distribution of the dipole angles for water molecules hydrating a central potassium or sodium cation (a) or chloride anion (b) within the first hydration shell of the ions. Data for the KCl- and NaCl-containing datasets are shown with solid and dashed lines, respectively. This angle is defined as the angle between the vector connecting the ion and the hydrating water molecule, and the vector corresponding to the dipole moment of the water molecule.

Combined, these results imply a difference in the hydration structures of the K and Na cations. Similarly to literature results, we find a tighter, more ordered distribution of water molecules surrounding the Na cation relative to that of the K cation which has a more disordered hydration structure, owing to the differences in ionic radii and charge density of the two ions.

With regard to the hydration of the chloride anions, both the chloride-water RDFs (Fig. S4) and the water dipole angle distribution in the first hydration shell (Fig. S5), it is evident that the structure of the hydration shell is largely independent of the cation, whether it be potassium or sodium. In both cases, the water dipole angle distribution has a maximum at 47.5°, as would be expected for a water molecule which is pointing its hydrogen atoms towards the hydrated ion and equivalent to that at the maximum of the dipole angle distribution for water molecules hydrating a TMG carboxyl oxygen atom in Fig. 9.

TMG hydration coordination numbers

The coordination numbers for hydration at the carboxyl oxygen (O) and methyl carbon (C_m) sites were calculated using the RDFs displayed in Figs. 7 and 8, and the resulting values are displayed in Table S5.

Table S5. TMG-water coordination numbers in the KCl- and NaCl-containing solutions. The standard deviation of the distributions are displayed in parentheses.

Environment	Coordination number	Cutoff r / Å
O-O_w (KCl)	2.67 (0.84)	3.3
O-O_w (NaCl)	2.70 (0.86)	3.3
C_m-O_w (KCl)	7.72 (1.55)	4.6
C_m-O_w (NaCl)	7.75 (1.57)	4.6

Ion coordination numbers

Running coordination numbers, i.e. as a function of radial distance, were calculated from the relevant pair distribution functions and Eq. S2 for various ion-oxygen interactions, and are displayed in Fig. S6. These were calculated for the TMG oxygen-cation (O-Z) interaction and the reciprocal Z-O interaction, shown in Figs. S6 (a) and (b), respectively. They were also calculated for the cation-water oxygen (Z-O_w) interactions (Fig. S6 (c)) and the total cation-oxygen coordination, Z-(O+O_w) (Fig. S6 (d)), calculated from the sum of Figs. S6 (b) and (c). In all of the figures, vertical lines illustrate the radial cutoff distances used to determine absolute coordination numbers in the first coordination shell; these were set to r = 3.5 Å for Z = K, and r = 3.2 Å for Z = Na, the distance at the first minimum in the relevant pair distribution functions. Due to the flatness of the running CNs around the radial cutoff values, the reported CN is largely independent of whether the specific O-K or O-Na cutoff distance is used. For example, if the O-K cutoff was used for the O-Na calculation, and vice versa, the difference in the resulting O-Z CNs is small ($\sim$ 0.02).

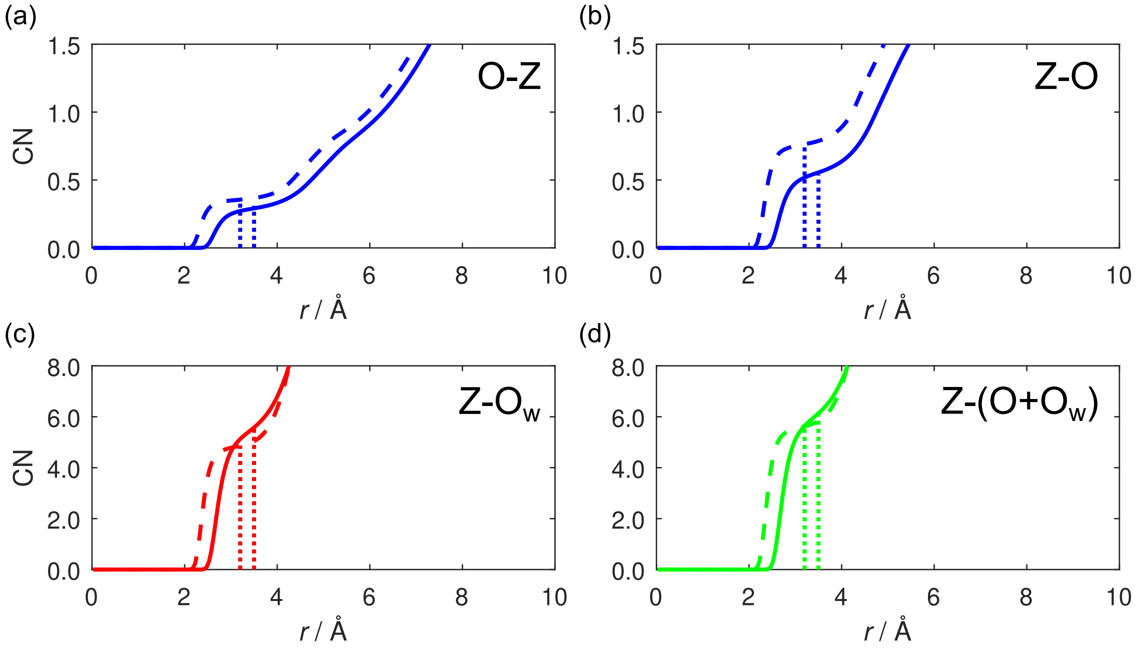

Figure S6. Running coordination numbers (CN) as function of radial disance r for ion-oxygen interactions, shown for potassium (solid line) and sodium (dashed line) ions. The functions are shown the following interactions: (a) O-Z, (b) Z-O, (c) Z-O_w, and (d) Z-(O+O_w). In each case, vertical dotted lines are shown at the radial cutoff values to illustrate the determined coordination number; r = 3.5 Å for Z = K, and r = 3.2 Å for Z = Na.

As we discuss in the main text, Fig. S6 (b) illustrates the enhanced coordination of TMG oxygens around sodium ions relative to potassium ions, along with the increased compactness of the coordination shell. The total coordination of oxygen atoms around the ions shown in Fig. S6 (d), from both TMG and water molecules, also reflects this difference. Whilst it may be expected that potassium ions should have a larger oxygen coordination number than sodium, it has been shown that these values decrease and converge at increasing ion concentration[5,8]. Additionally, the presence of TMG further increases the similarity of

these coordination numbers, as the bidentate carboxyl oxygens can sit closer together than two separate water oxygens ($\sim$ 2.1 Å compared to $\sim$ 2.8 Å), an effect more pronounced for sodium, as described above.

Bulk water structure

In this section we consider the structure of the water network in our solutions and the perturbations to the structure of pure water due to the presence of additional solutes. In Fig. S7 we show the RDFs that describe the correlations between water molecules in both of the KCl- and NaCl-containing solutions, as well as a comparison to literature results for pure water[9]. No differences between the calculated H_2O-H_2O RDFs are evident between the KCl and NaCl-containing solutions.

The O_w-O_w RDF (shown in blue) is indicative of the correlations between the centres-of-mass of water molecules, whereas the O_w-H_w considers correlations between the oxygen atoms of a reference water molecule and the hydrogen atoms of surrounding molecules and is informative about the hydrogen bonding structure between water molecules.

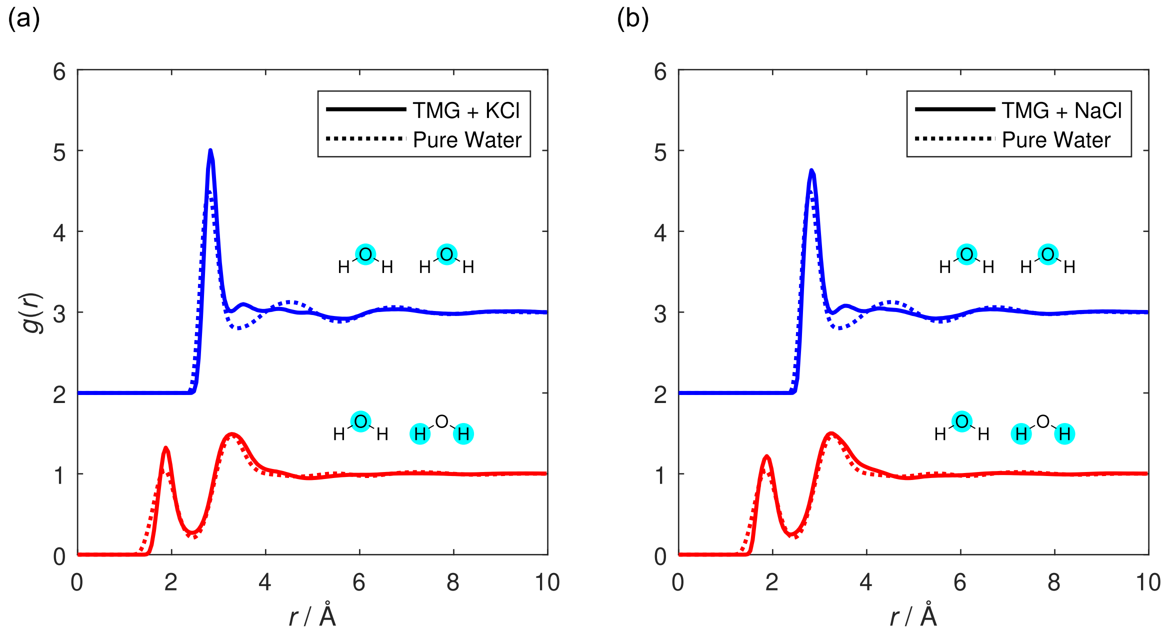

Figure S7. Intermolecular RDFs $g(r)$ for correlations between atomic sites in H_2O for: (a) the TMG + KCl solution and (b) the TMG + NaCl solution. In each case, the O_w-O_w (blue) and O_w-H_w (red) correlations are shown. The correlations present in pure water are shown as dotted lines, reproduced from Soper[9]. Only correlations between atoms in different water molecules contribute to the displayed functions. For clarity, the functions have been vertically shifted.

The primary peak in the O_w-O_w RDF is insensitive to the addition of the solutes - located at 2.8 Å in both pure water and our measurements - and is consistent with previous literature measurements of salt-containing solutions[6] and molecular osmolytes such as proline[10]. More notable is the disruption to the secondary peak, located at 4.5 Å in pure water but not discernable in the measurements of our solutions. A variety of effects upon this second O_w-O_w peak have been observed upon addition of solutes to aqueous solutions. In some instances, most notably upon the addition of monovalent salts[6,7], there is a marked inwards shift of this peak; to which an equivalence is made to the compression of water structure upon the application of a large external pressure[11,12]. A similar inward shift of this second peak was also observed

Table S6. Comparison of the water self-coordination numbers in the KCl- and NaCl-containing solutions. †The pure water coordination number is reproduced from Soper[9]. The standard deviation of the distributions are displayed in parentheses.

Solution	Coordination number	Cutoff r / Å
Pure water†	4.67	3.4
TMG + KCl	3.99 (1.19)	3.4
TMG + NaCl	4.10 (1.16)	3.4

by Gioacchnino *et al.* upon the addition of TMG on its own to an aqueous solution[13], in contrast to the observation made upon the addition of another osmolyte trimethylamine *N*-oxide (TMAO) which was able to resist these pressure or salt-induced perturbations to the structure of water, shifting this peak outwards to larger distances[14,15]. It is difficult to discern a second peak in our case, similar to the flattening of the peak observed for solutions containing other large solutes such as proline or *tert*-butyl alcohol[10,16]. Instead, we observe several small peaks following the large, first peak. A similar modification was observed in the O_w-O_w RDF of a water-in-salt electrolyte, where the first small peak was attributed to the vertex-vertex distance in the tetrahedral water structure[17]. Therefore, this peak arises from an inward shift of the second O_w-O_w peak (for pure water), but such modifications are ultimately caused by solutes occupying space and pushing nearest-neighbour water molecules to longer distances.

Consistent with these previous studies, we do not notice any significant changes to the O_w-H_w RDF relative to pure water upon addition of the solutes. This RDF has a primary peak at 1.9 Å, corresponding to the length of the hydrogen bonds between water molecules. Overall, this set of RDFs are indicative of a water hydrogen bond network that remains intact but is distorted due to an insufficient number of water molecules present to form a coordination shell beyond the first without other species also present.

Further characterisation of the bulk water structure can be performed by calculating the water dipole angle distribution of molecules around a reference water molecule ~~(Fig. S8)~~. In the context of the bulk water structure, one can calculate the distribution of water dipole angles hydrating a central, reference water molecule; this distribution is presented in Fig. S8 ~~(a)~~. It is clear that this distribution is bimodal: there is one peak centred on a dipole angle of 48.5° and a second, broader peak centred on 147.5°. This bimodal distribution is consistent with water molecules being both hydrogen bond-acceptors and donators, and would be expected for an analogous distribution for pure water.

The water self-coordination numbers, calculated using Eq. S2 using the O_w-O_w RDF and a cutoff of 3.4 Å are displayed in Table S6. The water self-coordination numbers are evidently reduced in the presence of solutes relative to pure water; the deviation can be attributed to the large size of the TMG molecules and the high concentration of both the TMG and salt, so an appreciable fraction of water molecules have one of these species in the first coordination shell, consistent with the lack of observable water-water secondary peak in Fig. S7.

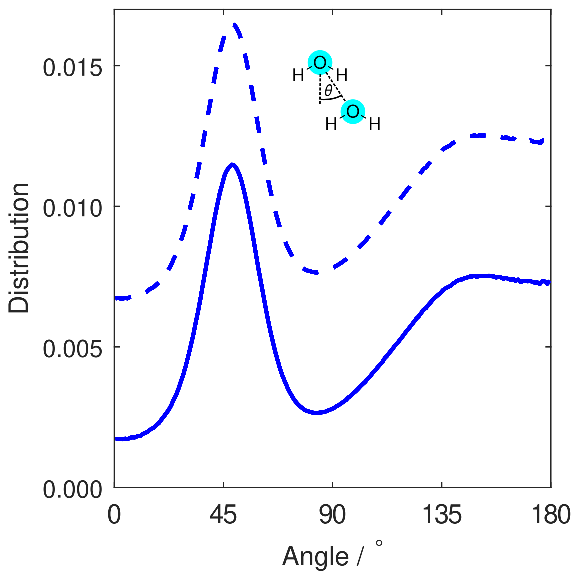

Figure S8. Distribution of the dipole angles for water molecules hydrating a reference water molecule. This angle is defined as the angle between the vector connecting the oxygen atom of a reference water molecule and the oxygen of a hydrating water molecule, and the vector corresponding to the dipole moment of the water molecule. Distributions are shown for the TMG + KCl (solid line) and TMG + NaCl (dashed line) solutions, which are shifted for clarity.

References

[1] Y. Wu, H. L. Tepper and G. A. Voth, *Journal of Chemical Physics*, 2006, **124**, 24503.

[2] W. L. Jorgensen and J. Tirado-Rives, *Proceedings of the National Academy of Sciences of the United States of America*, 2005, **102**, 6665–6670.

[3] L. S. Dodda, J. Z. Vilseck, J. Tirado-Rives and W. L. Jorgensen, *Journal of Physical Chemistry B*, 2017, **121**, 3864–3870.

[4] L. S. Dodda, I. C. D. Vaca, J. Tirado-Rives and W. L. Jorgensen, *Nucleic Acids Research*, 2017, **45**, W331–W336.

[5] R. Mancinelli, A. Botti, F. Bruni, M. A. Ricci and A. K. Soper, *Journal of Physical Chemistry B*, 2007, **111**, 13570–13577.

[6] A. K. Soper and K. Weckström, *Biophysical Chemistry*, 2006, **124**, 180–191.

[7] R. Mancinelli, A. Botti, F. Bruni, M. A. Ricci and A. K. Soper, *Physical Chemistry Chemical Physics*, 2007, **9**, 2959–2967.

[8] P. E. Mason, S. Ansell, G. W. Neilson and S. B. Rempe, *Journal of Physical Chemistry A*, 2015, **119**, 2003–2009.

[9] A. K. Soper, *ISRN Physical Chemistry*, 2013, **2013**, 67.

[10] S. E. McLain, A. K. Soper, A. E. Terry and A. Watts, *Journal of Physical Chemistry B*, 2007, **111**, 4568–4580.

[11] A. K. Soper, *Chemical Physics*, 2000, **258**, 121–137.

[12] A. V. Okhulkov, Y. N. Demianets and Y. E. Gorbaty, *The Journal of Chemical Physics*, 1994, **100**, 1578–1588.

[13] M. D. Gioacchino, F. Bruni and M. A. Ricci, *Journal of Molecular Liquids*, 2020, **318**, 114253.

[14] H. Laurent, T. G. A. Youngs, T. F. Headen, A. K. Soper and L. Dougan, *Communications Chemistry*, 2022, **5**, 1–10.

[15] H. Laurent, D. L. Baker, A. K. Soper, M. E. Ries and L. Dougan, *Journal of Physical Chemistry B*, 2020, **124**, 10983–10993.

[16] D. T. Bowron and S. D. Moreno, *The Journal of Chemical Physics*, 2002, **117**, 3753–3762.

[17] Y. Zhang, N. H. Lewis, J. Mars, G. Wan, N. J. Weadock, C. J. Takacs, M. R. Lukatskaya, H. G. Steinrück, M. F. Toney, A. Tokmakoff and E. J. Maginn, *Journal of Physical Chemistry B*, 2021, **125**, 4501–4513.
